# Approaches to Collect Comprehensive Electronic Patient Data Across Multiple Providers and Payers for Research: Landscape Analysis

**DOI:** 10.2196/86330

**Published:** 2026-06-15

**Authors:** Robert S Rudin, Nihar Chhatiawala, Joshua C Mandel, William J Gordon, David McCallie, Daniel Gottlieb

**Affiliations:** 1 Health Division, RAND Corporation, 20 Park Plaza, 9th Floor, Suite 910, Boston, MA, 02116, United States, 1 6173382059 ext 8636; 2 Health Division, RAND Corporation, Santa Monica, CA, United States; 3 Microsoft Research, Redmond, WA, United States; 4 Computational Health Informatics Program, Boston Children's Hospital, Boston, MA, United States; 5 Department of Biomedical Informatics, Harvard Medical School, Boston, MA, United States; 6 Department of Medicine, Brigham and Women's Hospital, Boston, MA, United States; 7 Formerly with Cerner Corporation, Kansas, MO, United States; 8 Central Square Solutions, Boston, MA, United States

**Keywords:** research data, interoperability, health information policy, landscape analysis, subject matter expert, SME

## Abstract

**Background:**

The digitization of medical data and advances in interoperability have opened opportunities for research studies to use more comprehensive, longitudinal patient data from multiple sources. As patients often interact with many providers and payers over time, collecting data across organizations may have critical implications for accuracy and bias in study results. US policy has promoted exchanging health information among providers, payers, and patients, but less attention has focused on facilitating data collection for research, which presents unique challenges.

**Objective:**

This study aimed to identify and evaluate existing and emerging approaches for collecting comprehensive provider and payer data for research in the United States, with the goals of informing researchers of possible methods and generating evidence to inform policy initiatives. Our focus was on electronic approaches to data aggregation for studies requiring patient consent.

**Methods:**

We conducted a landscape analysis through interviews with subject matter experts (SMEs). SMEs were selected based on expertise. We created a list of evaluation criteria, identified existing and emerging approaches, and described the benefits and limitations of each approach by applying the evaluation criteria. We interviewed SMEs until saturation was achieved. Data collection was limited to the United States.

**Results:**

A total of 20 SMEs helped identify 8 distinct approaches: (1) general-purpose smartphone app, (2) commercial app, (3) research community app, (4) structured data export, (5) Trust Exchange Framework and Common Agreement Individual Access Service, (6) regional study query, (7) national study query, and (8) aggregated data source. Participant-mediated exchange approaches (1-5) leveraged patients’ right of access. Three approaches leveraged existing data exchange services (5-7). To evaluate these approaches, we identified 12 criteria, including perspectives of participants, research teams, and broader stakeholders. Each approach had benefits and limitations; no single approach emerged as superior for all use cases. While currently available approaches for participant-mediated exchange bypass the need for complex governance arrangements, they are limited by participant burden, effort needed by research teams, and data gaps, especially from payers. Some regional health information exchanges and aggregated data sources address governance challenges and can provide services such as preparing analytic datasets but are restricted to specific locations and/or data-source coverage. National networks currently do not allow queries for research and confront challenges in establishing trust and enforcing compliance with data-sharing requirements among network sites.

**Conclusions:**

Collecting comprehensive health data from multiple providers and payers in the United States is a complex and evolving process. The suitability of an approach may vary based on the needs of a study. Given the numerous barriers and the lack of a clear dominant method, further exploration and benchmark comparisons of all identified approaches are necessary. Ongoing public policy efforts will likely play an important role in progress.

## Introduction

### Overview

The large-scale digitization of medical data and widespread adoption of electronic health records (EHRs) in the United States provide researchers with the opportunity to use longitudinal data on participants from across their sites of care [1,2]. Yet, today, typical health care research efforts in the United States lack access to complete patient data [3-6]. US policy efforts, such as the 21st Century Cures Act and related regulations, have promoted approaches for exchanging health information among providers and payers, and with patients, including for purposes of facilitating data collection for research purposes [7-11]. To better understand existing and emerging approaches, we conducted a landscape analysis with the aim of informing researchers and policy efforts.

### Background

The capacity to conduct studies that extend beyond a single institution and are not confined to a limited network of health care providers is crucial. Without access to comprehensive data, researchers may overlook important information regarding participants’ prior health-related encounters, potentially resulting in biased outcomes [3-6]. The absence of complete data can also impede the feasibility of some kinds of studies, including those aimed at evaluating the long-term value of health care and those seeking a holistic understanding of participants’ health [12].

Data for most patients are fragmented across multiple organizations. In the United States, for example, a typical Medicare beneficiary sees a median of 2 primary care physicians and 5 specialists working in 4 different practices [13]. Beneficiaries with multiple chronic conditions have even greater levels of fragmentation. Studies that recruit patients of only 1 primary care physician will find that, on average, every 100 of their Medicare patients receive care from 53 different practices [14]. The effort required to access and link these data can be prohibitively large, even for studies enrolling only a small number of patients. Payer data also require substantial effort to collect. Patients may change health plans during a study period—one study found that 21.5% of members turned over each year [15]—further adding to the fragmentation and effort needed to assemble complete data.

The goal of assembling longitudinal patient data has long been recognized [2,16]. However, previous attempts to achieve this goal in the United States have failed. For example, one effort to create a patient-controlled “health record bank” closed due to limited enrollment [17]. Some health information exchange (HIE) organizations have assembled data from multiple health care providers within geographic regions, but many face challenges with sustainability [18]. National data exchange networks focus primarily on clinical care; their potential to facilitate data collection for research has not been examined.

We interviewed experts to identify the range of possible approaches that research teams might use to collect multi-institutional, comprehensive provider and payer data on patients enrolled in studies and to evaluate their benefits and limitations. By assessing the current landscape, this work ultimately aims to help research teams select among the options available to them for collecting data on their studies’ enrolled patients, help research teams prepare for emerging options, and inform efforts to further facilitate the complete collection of patient data for research.

## Methods

### Objective and Scope

Landscape analyses aim to comprehensively describe and map the current state of a domain of interest and provide an overview to inform decision-making [19-21]. This approach is particularly useful for rapidly evolving domains where formal literature may not reflect current practice or emerging innovations. This US landscape analysis had three objectives:

1. Identify evaluation criteria for assessing approaches that research teams might use to collect comprehensive provider and payer data on study participants.
2. Identify existing and emerging approaches to the problem.
3. Evaluate the benefits and limitations of the identified approaches by applying the evaluation criteria where applicable.

We focused on approaches that would support research studies in which patients provide consent for their data to be collected from multiple sources and used for research. This would include many kinds of clinical trials, implementation studies of clinical interventions, and observational studies. Approaches that do not require patient consent (eg, using data from a deidentified repository for population-level studies), involve a single source of data (eg, from one payer or one provider), or only make a narrow subset of the medical record data available (eg, clinical registries) were not included, as they would involve different research workflows.

We included health care providers and payers as data sources only because of their prevalent use in health care research studies; other sources (eg, wearables, social media, community-based organizations, and direct-to-consumer genetic testing services) were not included. We also excluded imaging data (eg, magnetic resonance imaging) because of differences in the technology and workflows needed to access these data compared with other data derived from health care encounters. We included approaches that addressed the following activities:

- Identifying the source and location of the data: determine where the participant previously received care or may have electronic health data available, including through patient self-identification, following chains of records (eg, from a payer to the providers that billed for care), and query tools that access metadata describing data sources (eg, name, address, Fast Health Interoperability Resources [FHIR] endpoint) and match using demographic characteristics.
- Retrieving the data: collect electronic health data from the data source through manual or automated download processes and the use of specific software tools to facilitate the data collection.
- Sharing the data with research studies: make electronic health data available to research team members, including automated approaches and those that require an extra step on the part of an individual research participant.

Approaches to identify patients for eligibility or recruitment into studies required separate research workflows and were out of scope.

We limited our scope to electronic approaches, particularly those that retrieve data using application programming interfaces (APIs) such as FHIR, because of their potential to facilitate data collection; paper- or fax-based approaches were considered out of scope. Additionally, only approaches that were considered by the research team or experts to be reasonably feasible for implementation within the next few years were included.

### Recruitment of Subject Matter Experts and Interviews

We identified subject matter experts (SMEs) through publications, recommendations from our professional network, and recommendations from other SMEs (ie, snowball sampling). We conducted each interview via videoconference and recorded all interviews, which lasted roughly 1 hour. If we had additional questions, we requested a second interview with the SME or exchanged follow-up emails. In some cases, 2 SMEs attended the same interview. Interviews were conducted between February 2024 and July 2025.

To prepare for the interviews, we prepared preliminary lists of approaches and evaluation criteria, and a table applying the evaluation criteria to each approach. The research team developed these based on its existing knowledge of the landscape. We also prepared an interview guide that covered our 3 objectives. Specifically, we asked SMEs to identify and describe in detail all existing and emerging approaches with which they were familiar for collecting provider and payer data for research, identify evaluation criteria for assessing these approaches, and evaluate the approaches based on the evaluation criteria. As SMEs had expertise with different approaches, we focused the discussion on approaches with which the SME was most familiar. For SMEs with broader knowledge of the landscape, we reviewed our preliminary lists of approaches and evaluation criteria, asked them to identify anything missing, and asked them about what policy or other health care system changes may influence the assessment of approaches. Finally, we asked the SMEs to identify relevant publications or other documents that should be reviewed to understand the evolving landscape of approaches. After each interview, we updated and refined our preliminary lists of approaches, evaluation criteria, and application of evaluation criteria to approaches. We continued interviewing SMEs until we achieved saturation in terms of evaluation criteria and approaches. We aimed to include at least 1 SME with detailed knowledge of each identified approach.

### Analysis

We used conventional content analysis to analyze the qualitative data [22,23], inductively identifying approaches, evaluation criteria, and applications of evaluation criteria to the approaches. For each interview, one research team member summarized key points discussed and extracted relevant quotes, and a second research team member reviewed each recording and the summaries and notes (RSR and NC). Both research team members had domain experience with EHRs and digital health. Differences were resolved by consensus. To generate overall findings, after each interview, we updated a working matrix that mapped evaluation criteria against approaches, noting areas of agreement, disagreement, and uncertainty across SMEs. For evaluation criteria, we compiled all factors mentioned by SMEs, grouped conceptually similar factors, and refined descriptions through discussion until we achieved a comprehensive list, aiming to exhaustively cover all important factors for assessing the benefits and limitations of the various approaches. Evaluation criteria were not required to be mutually exclusive. For approaches, we created detailed written descriptions that included technical details, workflows, and perspectives from relevant interviews. We then applied each evaluation criterion to each approach, synthesizing assessments from SMEs. We noted where SMEs provided conflicting assessments or expressed a lack of sufficient evidence or experience to make an assessment.

To ensure rigor and trustworthiness of our findings, we used the following methods during the analysis: 2 researchers independently reviewed recordings and summaries, resolving discrepancies through consensus discussion; SMEs were selected to include a range of perspectives (eg, technology developers, policy experts, and researchers); we reviewed our preliminary findings with SMEs who had broad knowledge of the landscape; and we continued sampling until we achieved saturation. Additionally, all authors have relevant domain expertise in EHRs, interoperability, and digital health, and contributed to interpreting technical content and synthesizing results.

We strengthened the rigor of our findings and supplemented our interview data by incorporating 3 types of documents suggested by SMEs into this analysis. First, we reviewed technical documentation (eg, FHIR implementation guides and Trust Exchange Framework and Common Agreement [TEFCA] policies) to verify and elaborate on technical details described by SMEs. Second, we reviewed gray literature sources (eg, blog posts and white papers) to understand emerging perspectives. Third, we reviewed relevant peer-reviewed publications to contextualize SME assessments. These documents helped confirm factual claims and provide supplemental information.

### Ethical Considerations

This study was reviewed and approved by both the RAND Human Subjects Protection Committee and the Mass General Brigham Institutional Review Board (IRB) as exempt. All written and audiovisual data were stored on secure servers accessible only to the research team. Verbal consent was obtained before conducting the interviews, which included permission for identifying the individual in the publication but not attributing any quote or information to any specific individual. We chose to identify SMEs to enhance transparency and allow readers to evaluate the credibility and relevance of our sources [24]. Participants were not compensated.

## Results

### SME Characteristics

We interviewed 20 SMEs (Table 1). SME expertise covered a wide range of aspects relevant to this topic, including smartphone-based approaches, FHIR, and federal and state policy.

**Table 1.** Names and characteristics of subject matter experts interviewed in the study.

Subject matter expert	Title (at time of interview)
Ricky Bloomfield	Chief Medical Officer at ŌURA; formerly Clinical and Informatics Lead at Apple
Dan Drozd	Chief Medical Officer at PicnicHealth
Andrew Cress	Chief Executive Officer and Cofounder at HealthVerity
Andrew Goldberg	Chief Operating Officer and Cofounder at HealthVerity
Ryan Howells	Lead of CARIN Alliance; Principal at Leavitt Partners; digital health policy and interoperability expert
Jocelyn Keegan	Program Manager of the HL7 Da Vinci Project; Payer-Practice Lead at Point-of-Care Partners
Brenden Keeler	Head of Product at Flexpa
David Kendrick	Chief Executive Officer of MyHealth Access Network, the Oklahoma state-designated entity for health information exchange
Vik Kheterpal	Principal at Care Evolution, Inc
Mark Knee	Director, Interoperability Division, Office of the National Coordinator for Health Information Technology
Greg Liptak	Vice President for Research and Innovation at HealthShare Exchange, the regional health information exchange for greater Philadelphia
Joel Montavon	Clinical Informaticist at RTI International
Michele Mottini	FHIR^a^ software developer at Care Evolution, Inc
Christopher Muir	Director, Network and Scalability Division, Office of the National Coordinator for Health Information Technology
JP Pollack	Cocreator of CommonHealth; Cofounder of The Commons Project Foundation
Molly Prieto	Deputy Director, Standards Division, Office of the National Coordinator for Health Information Technology
Marc Rabner	Chief Medical Officer of Chesapeake Regional Information System for Our Patients (CRISP) Shared Services
Raheel Sayed	Lead of the People Heart Study, which uses Apple Health
Jeff Smith	Deputy Director, Certification and Testing Division, Office of the National Coordinator for Health Information Technology
Mariann Yeager	Chief Executive Officer of the Sequoia Project, which supports TEFCA^b^

a FHIR: Fast Health Interoperability Resources.

b TEFCA: Trust Exchange Framework and Common Agreement.

### Evaluation Criteria for Assessing Approaches

We achieved saturation by identifying 12 evaluation criteria (Table 2). We took an inclusive approach and included even those criteria that might be challenging to apply.

**Table 2.** Evaluation criteria for assessing approaches to collect comprehensive provider and payer data on study participants.

Evaluation criterion	Description
Reach and accessibility	Volume of participants whose data can be accessed (includes requirements for devices such as smartphones).
Data availability and quality	Extent to which records and their contents are available and usable for research (includes consideration of whether variables are in designated fields, completeness due to factors such as geographic coverage and accuracy).
Study team burden	Effort or cost required to implement and operate a fully developed approach (includes required technical support).
Feasibility	Effort or changes required to make the approach widely available (includes technical effort needed by stakeholders).
Participant burden	Effort or cost required of participants to use the approach.
Participant benefit	Potential benefits that might motivate use.
Security	Risk or perceived risk of breach.
Privacy	Nature of patient control over data.
Impact of industry trends	Potential for technology or other trends to affect the approach (eg, adoption of technical standards).
Sensitivity to changes	Potential for stakeholders to make decisions that affect the approach (eg, market changes in service providers).
Alignment and synergies with policies and policy goals	The extent to which the approach supports or is supported by policies and policy goals and lacks regulatory barriers.
Configurability and innovation enablement potential	Potential for the approach to foster innovation in research and practice (eg, customizability).

### Evaluation of Approaches to Collect Comprehensive Data for Research

We identified 8 approaches, which achieved saturation within the scope of the study (Table 3 contains summary assessments; approaches are labeled 1 through 8; Multimedia Appendix 1 contains full application of all evaluation criteria to all approaches). Five are participant-mediated approaches (1-5), in which research participants are directly involved in retrieving their data. In the remaining approaches, which included study queries of existing data exchange infrastructure (6 and 7) and aggregated data sources (8), participants’ roles were limited to providing consent. Many SMEs emphasized the benefits of participant-mediated approaches because they leverage patients’ right of access to their own data. Four of those approaches required study participants to manually identify their sources of data and use the patient or member portals at each site (1-4). In contrast, the other approaches leveraged existing national qualified health information networks (QHINs), regional HIEs (also called regional health data utilities), or aggregated data sources to identify sites of care and pull data. For these latter approaches, SMEs stated that although providers and payers, as Health Insurance Portability and Accountability Act (HIPAA)–covered entities, are legally required to release data with patient consent, rigorous governance processes are often required because of concerns about liability from errors and the lack of financial incentives to streamline data sharing.

**Table 3.** Approaches that research teams might use to collect comprehensive provider and payer data on study participants and summary assessment.

Approach name	Description	Summary assessments^a^
Participant-mediated approaches		
General purpose smartphone app	Participants use Apple Health (iOS) or Common Health (Android) to collect data and share it manually or through a purpose-built study app	Benefits: widely available as an installed app (Apple Health) or an installable app (CommonHealth); management of FHIR^b^ endpoints is handled by the app. Limitations: participant burden for manual data sharing or research team burden to create and maintain a study app, not all FHIR resources may be supported, gaps in FHIR endpoint coverage, potential perceived privacy concerns, limitations in customizability, and potential sampling bias due to smartphone requirement.
Commercial app	Study pays for an app allowing participants to collect and share data	Benefits: management of the FHIR endpoints directory handled by the app, processing of data can minimize study team burden of analysis, additional features of value offered to patients, and some apps have the potential to be accessed from a web browser. Limitations: cost to the research team, potential perceived privacy concerns, and dependency on the app company for customizations.
Research community app	A study or institution uses a homegrown or open-source app allowing participants to collect and share data	Benefits: potentially lower cost for the research team, reduced perceived privacy concerns, potential for additional features of value to patients, and potential for access from a web browser. Limitations: requires development effort to become widely available and is a burden on the study team or organization to operate and to manage the FHIR endpoint directory and client registrations.
Structured data export	Participants use EHI^c^ export or a similar feature to collect data	Benefits: may become widely available in the near future, likely accessible from any web browser, potential reduced perceived privacy concerns, and potentially be comprehensive in terms of data. Limitations: not widely available today, burden on the participant to manually download and share data, burden on the study team to set up a process to receive data shared by the participant, and lack of data format standardization.
TEFCA^d^ Individual Access Service	Participants access data directly from a national network (QHIN^e^)	Benefits: participant burden is minimal because they would need to use only one app or portal and is supported by current policies. Limitations: not usable today, burden on the study team to set up a process to receive data shared by the participant, and participants may still need to log into sites of care.
Queries of data exchange infrastructure		
Regional study query through a HIE^f^	Participants consent for the study team to query an HIE	Benefits: minimal or no burden on participants and processing of data can minimize the study team’s burden of analysis. Limitations: unavailable today in most regions, gaps in data coverage, especially among payer claims, and cost to the research team.
National study query through QHINs	Participants consent for the study team to query a QHIN	Benefits: minimal or no burden on participants. Limitations: research is not currently an authorized exchange purpose, and the timeline for adding it is uncertain (although it is aligned with policy goals); there may be gaps in QHIN coverage of sites, the burden on the study team is unknown, and potential perceived privacy concerns.
Use of aggregated data sources		
Aggregated data source	Participants consent for the study team to collect data from a company or organization that has already collected data from multiple sources	Benefits: minimal or no burden on participants, data sources are nationwide, and processing of data can minimize the study team’s burden of analysis. Limitations: may not cover all data sources or elements needed for the study and cost to the research team.

a Summary assessment is based on the criteria listed in Table 2 and includes selected key findings.

b FHIR: Fast Health Interoperability Resources.

c EHI: electronic health information.

d TEFCA: Trust Exchange Framework and Common Agreement.

e QHIN: qualified health information network.

f HIE: health information exchange.

### Approaches Using Patient and Member Portals

Three of the approaches we identified required study participants to access their patient and members’ portals to retrieve data using Substitutable Medical Applications and Reusable Technologies (SMART) on FHIR (Table 3; approaches 1-3). To avoid repetition, we describe the assessment of this component here. Benefits of this approach include the high availability of patient and member portals. Patient portals have become increasingly used since the COVID-19 pandemic and are required to support patient access to data via SMART on FHIR, which addresses security concerns and allows patients some granular control over their data. Payer member portals are also widely available, although usage rates are not known. Additionally, these approaches align with federal policy goals. Facilitating patient access to their own health data has been a primary policy objective and a driver of certification programs and functionality for many years [25,26] and continues to be a priority [27]. Some SMEs pointed to more recent plans by Centers for Medicare & Medicaid Services (CMS) to advance digital identity solutions in health care and were optimistic that substantial progress toward the use of single sign-on (SSO) to patient portals, and potentially member portals, was occurring as complex issues such as price transparency and antitrust issues were being addressed [26,28].

There are 2 limitations to relying on patient and member portals for collecting data for research. First, the burden on study participants to log in to each portal is substantial. Patients may not remember prior locations of care, may have trouble finding login information, or lack portal access at some locations, and may be unwilling to spend time registering. This may result in incomplete data. However, adoption of SSO technologies could reduce this barrier substantially and is part of the CMS Interoperability Framework [29-31]. Additionally, studies may need to rely on participants for the timing of data retrieval, which may introduce variability.

Second, approaches that rely on patient and member portals must register with FHIR endpoints and establish a searchable directory for participants. The process of registering varies by EHR vendor or, in many cases, by individual site. One SME described the challenges: “It’s kind of a nightmare. We rely on a combination of reaching out to providers where the data is really messy and communicating with the EHR vendors who are only partially motivated to have that information be correct.” One SME estimated that “maybe about 10% of providers actually care about this… make sure that [metadata] is correct.” A recent FHIR standard called “User-access Brands and Endpoint” is designed to help users locate their data sources but has not been widely adopted, although recent regulation requires certified EHR vendors to make endpoint lists available in FHIR format [32,33]. Support from vendors and providers is also variable: “Sending email into the void is a common thing.” Some vendors have needed to develop automated tools and dedicate teams to monitoring endpoints. Recent CMS commitments to “building a dynamic, interoperable national provider directory” may help address these barriers [34].

Payer member portals have their own unique challenge, such as a lack of compliance with FHIR standards, missing data fields, delays in making data available, and technical issues resulting in downtime. SMEs noted that only CMS-regulated plans are required to support individual access to payer data, and only a minority of commercial plans have opted to offer this service voluntarily, though several states have begun efforts to extend this requirement to all payers. Some SMEs suggested that, for payers operating both commercial and CMS-regulated plans, it would be technically easier to offer all their members access to their data, but some are investing substantial resources to make this service available only to members with plans that require it. Even for those that require it, SMEs suggested that many payers were not investing sufficiently, resulting in poor service: “They were more concerned about compliance and getting penalized. And now that they have done a shoddy job for 2 years and nothing has happened, they have developed comfort to say ‘whatever.’” SMEs hoped that when payers are required to share their data with other payers, they will also make that data available to individuals because the same FHIR endpoints would be used: “It is one thing when 5 or 6 app makers call you up and complain that your API is non-conformant but when 300 payers call you and complain I think it will probably be a different story.”

### General-Purpose Smartphone App

#### Description

Study participants can use general-purpose smartphone apps to pull their data from patient or member portals, store that data in the apps on their smartphones, and share the data with research teams. Both major smartphone operating systems allow for this functionality. On iOS, the Apple Health app enables users to retrieve their health records and store them on the device in Apple’s HealthKit [35]. On Android, a third-party app called CommonHealth enables users to retrieve their health records and store them in the app or in the device’s HealthConnect repository [36]. In both cases, users retrieve their data by logging into patient and member portals using SMART on FHIR protocols. However, the FHIR endpoint directory functionality, which allows users to search for their providers and payers, is different: in iOS/Apple Health, providers must register with Apple and provide metadata, including a logo, and payers are not supported; in Android/CommonHealth, a larger directory of providers and payers is supported, and sites do not need to register. To share data with research studies, both iOS/Apple Health and Android/CommonHealth offer APIs that allow purpose-built apps to integrate with these tools, and there are some open-source examples available [37,38]. Absent a purpose-built app, users could manually share an export of their data for iOS/Apple Health by uploading it to a study server, or create a clinical summary using Android/CommonHealth and allow the research team to access it with a 6-digit passcode [39], but these features were not designed for the research use case and would be more burdensome for participants compared with a purpose-built app.

#### Assessment

Refer to the “Approaches Using Patient and Member Portals” subheading for the benefits and limitations of that component of this approach. We found benefits and limitations in the way Apple Health and CommonHealth managed FHIR endpoint directories: Apple Health’s directory excludes all payers and many providers but may have more accurate and usable metadata to allow for easier search, whereas CommonHealth’s includes payers and a larger number of providers, but the metadata is not curated. There are also differences in available data—for example, only CommonHealth includes the FHIR Patient or Encounter resources. Both projects’ prior conformance testing on the FHIR data helped identify issues that the site could address to make the data more usable for research and other purposes. One benefit of this approach is that these data, once collected, have the potential to also be used by participants for self-care health apps [40].

Limitations of this approach identified by SMEs include the burden on the research team to establish a mechanism for participants to share their data with the research team—either a burdensome manual process or the development of a custom app; the perception of privacy concerns among participants regarding having their data accessible to technology companies without any affiliation with their health care; dependency on the company for functionality and customizations (eg, inclusion of payer data); and possible sampling bias due to the requirement that participants own and use a smartphone.

#### Commercial App

##### Description

SMEs identified several companies that offer products designed to help facilitate research participants sharing their electronic health data with research teams [41]. These products allow users to retrieve their data by logging into their patient and member portals using SMART on FHIR protocols. Some use other, less standardized methods for retrieving the data. The companies that develop these products curate site metadata for their FHIR endpoint directories. Some products are browser-based and therefore not tied to smartphones. We found some products that targeted specific niches within this area, such as focusing exclusively on payer data or supporting clinical trials sponsored by pharmaceutical companies.

##### Assessment

Refer to the “Approaches Using Patient and Member Portals” subheading for the benefits and limitations of that component of this approach. Unlike approach 1, these products are not limited to smartphones and therefore have a broader reach. Because these products are designed specifically for collecting data for research, they minimize burden on the study team and provide technical support, but they come with a financial cost. Some products provide features that may be helpful depending on the study’s needs, such as converting a FHIR payload to the Observational Medical Outcomes Partnership Common Data Model [42] or identifying gaps in data (eg, data elements that would be expected for a study of early breast cancer) to inform the need for further data collection. Participants who use these apps may benefit from other features offered by them that leverage their aggregated health data [40].

Limitations include study participants’ potential privacy concerns with sharing data with a commercial company with which they have no direct relationship. However, the extent of this concern is unknown. An SME from one of these companies said, “We wouldn’t have a business if we weren’t able to build and maintain that trust with patients.” Commercial apps also may have limitations in their ability to be customized for specific study needs.

### Research Community App

#### Description

We identified two noncommercial software tools developed to facilitate data collection for research studies: Sync-for-Science Procure [43] (developed by co-author DG and available as open-source) and Wellmine [44] (developed by RTI International and available for studies conducted by that organization). This approach allows users to retrieve their data by logging into their patient and member portals using SMART on FHIR protocols. Research studies, or their institutions, using these products would need to deploy an instance of the product and create and manage their own FHIR endpoint directories, registering the instance with each endpoint. As with several of the commercial apps (approach 2), both of these tools are browser-based.

#### Assessment

Refer to the “Approaches Using Patient and Member Portals” subheading for the benefits and limitations of that component of this approach. Benefits of this approach compared with approach 2 (commercial apps) include potentially reducing dependency on an external company, the potential for reduced privacy concerns if the product is viewed by participants as affiliated with a trusted organization (eg, their health care provider), the potential to more easily add customizations for study-specific needs, and the potential to foster a community to innovate in this area, which may involve developing features that provide value to patients from their aggregated health data. Limitations include the lack of polish that’s often present in open-source or homegrown software products, which may necessitate additional development, and the burden on studies or their institutions to establish and maintain an instance and FHIR endpoint directory; however, once established, a single instance of the app may be used across many studies.

### Structured Data Export

#### Description

In this approach, the study participant logs into each of their patient and member portals, downloads their health data onto their local device, and then shares it using a mechanism set up by the research team (eg, uploading the data to a study server). EHR vendors are required by the 21st Century Cures Act Final Rule to support electronic health information (EHI) export functionality for patient data [45]. The participant would need to request these data and then share them through a method established by the research team. EHI export may also be requested separately from a patient portal, such as by submitting a request form or email; such manual approaches are out of scope for this landscape analysis.

#### Assessment

As with other approaches that rely on the use of patient and member portals, there is a burden on study participants to remember their sites of care and health plans, establish portals, and log into each one, which may result in incomplete data. There may also be data completeness issues due to relying on the participants for the timing of the data sharing.

EHI export has generally not been made directly available to patients through portals, but SMEs suggested that it would become more available if requested by patients, as providing this capability is required under existing regulations to be supported by EHRs. Although the data may be more comprehensive compared with data available through FHIR-based approaches, it may be challenging to work with [40] because of nonstandardized data formats that vary by EHR vendor and site of care: “We left it up to developers to determine for themselves how to export information. We basically said the EHI export has to come with a data dictionary so people can figure out how to kind of piece things back together. But I have no doubt that there are some developers who do a fairly nice job with it and then other developers who do not.” Similar to EHI but less standardized, many portals currently allow patients to download some or all of their data in PDF format, which may be increasingly usable for research because they can be processed into structured data by large language models.

This approach does not involve SMART on FHIR and therefore no FHIR endpoint directory is required, reducing related study burden and/or costs. However, the study would still need to establish a method to allow for secure sharing of the data, and the manual effort required on the part of study participants may be challenging for many participants.

### TEFCA Individual Access Service

#### Description

In this approach, participants would use the Individual Access Service (IAS) required by the TEFCA [7]. TEFCA is designed to create a “network of networks” to facilitate the exchange of health information [7]. The IAS allows apps to register with participating QHINs (a designated network under TEFCA) and query for and aggregate all of an individual’s health data across QHINs. The data would then be shared with research studies either using functionality offered by the app or by asking the participant to export the data and share it using a method established by the research team (eg, uploading the data to a study server). This approach would require an app and, therefore, would need to be bundled with approach 1, 2, or 3.

#### Assessment

Benefits of this approach are that it would leverage TEFCA’s mechanism for searching for and retrieving individuals’ health records, obviating the need for research teams and app companies to establish FHIR endpoint directories; facilitate identification of data sources by using TEFCA’s record location and identity matching methods so that research participants would not have to remember their sites of care; potentially offer participants additional functionality based on the aggregated data; provide privacy controls if offered by the app; and align with existing policy supporting individual access to health data. Currently, some QHINs require users to authenticate with and approve an access request within the patient portal of each potential site of care to ensure correct patient identity matching and consent when using IAS; while the QHIN can still serve as a record locator, this greatly limits the benefits of this approach by adding burden to the patient. At other QHINs, patients may only be matched to sites of care that have a very high level of confidence in the accuracy of the match, potentially excluding sites of care with patient data. Active efforts are trying to increase support for shared login credentials across sites [31]. Apps that use the IAS must meet HIPAA privacy and security requirements through their signed participation agreements with QHINs (even though they are not obligated under HIPAA as covered entities) [7]. Additionally, apps that adhere to the CARIN code of conduct may increase the security safeguards even further [46].

The major limitation of this approach is that although it is technically available and required as part of TEFCA today, sites that receive IAS requests often refuse to respond to them. SMEs believed this was because sites do not believe that the data would always be released to the correct patient and that they would be blamed for HIPAA violations if the query resulted in an improper disclosure. One SME said that providers would not share records “until they know darn good and well that they’re not going to get sued for giving the wrong person the wrong information.” SMEs suggested that the adoption of FHIR-based queries would allow for more individual identity data to be used for record matching, thereby reducing the odds of improper disclosures. However, some SMEs believed competitive concerns were also a factor—sites did not want to share data with entities that did not also provide data back into the network. SMEs suggested that some method of enforcement would be needed. Use of SSO across patient and member portals may help with matching concerns by standardizing and improving the quality of identity information [29] as well as automating portal logins [47]. Other limitations include the lack of some data sources available on QHINs (eg, payers and many small EHRs do not participate), performance issues when trying to query large geographic areas, imperfect record matching resulting in missed data, lack of analytic dataset preparation (unless supported by the app), and lack of support for querying directly from an app on an individual’s device, which would be more privacy preserving.

### Regional Study Query Through HIE

#### Description

HIEs exchange electronic health data for multiple purposes within a specific geographic region or at a statewide level [48,49]. There is a high level of interest among HIEs in supporting research, but only some currently do so [50,51]. One barrier is that some HIEs that did not initially include research use cases are required to update their member participation agreements, which can be a cumbersome process. In this approach, the research team works with the HIE staff to specify their data needs for consented patients under an agreed-upon scope of work, which may include preparing the data in a specific format, for a fee. HIEs’ governance processes address state and federal legal concerns (eg, the sharing of sensitive health data) as well as issues raised by HIE members. The processes differ across HIEs. Some require IRB approval from several member organizations; others use a single IRB. Some allow access to claims data from multiple payers; others lack claims data. Some share clinical notes for research; others do not. Members may have the option to opt out of sharing their data for specific requests. There is an interest among at least some HIEs in providing patients with access to a portal for managing their consents for research projects. The HIE staff create the datasets and work with the research team to resolve issues, such as fixing demographic information if no record matches are found for specific patients.

#### Assessment

This approach would be widely accessible to all participants regardless of their ability to use technology, involve minimal burden on the participant, allow research teams to control the timing of the queries to reduce variability in retrieved data, broadly align with policy goals supporting data exchange, and may foster innovation in how HIEs can better support research needs. It leverages years of investment in infrastructure and governance processes in many communities. Some HIEs also include social determinants of health data from community-based organizations, which would be valuable for many research studies. Another benefit that SMEs pointed to is that HIEs can ensure compliance with state laws for data sharing centrally, which may be much more challenging for national-level queries. The major limitation of this approach is that it is currently available only in a limited number of geographic areas, though rigorous data are not available. Also, not all HIEs include claims data, and very few have access to Medicare or Medicaid data; their coverage of providers varies, and some states require patient opt-in, resulting in less available data.

### National Study Query

#### Description

In this approach, the participant consents to allowing the research team to query a national network of health care providers, such as CareQuality, Common Well Health Alliance, or other QHINs participating in TEFCA. The research team then performs the query and assembles the data. For these networks, queries are required to specify the “purpose of use” of the data [7]. Most queries today specify “treatment,” which requires that the querier be a provider of care and offer data back to the network for others to query (ie, reciprocity). Currently, participants are not required to accept requests that specify “research” as a purpose of use under TEFCA or any national network. SMEs described preliminary conversations among policymakers about making research a purpose of use in TEFCA, including plans to conduct a review of the landscape of laws across states and to gather other information from stakeholders.

#### Assessment

This approach would be widely accessible to all participants regardless of their ability to use technology, involve minimal burden on the participant or the research team, allow research teams to control the timing of the queries to reduce variability in retrieved data, and broadly aligns with policy goals. FHIR resources subscription standards under development could allow research teams to receive automatic updates for new data, which may be useful to some studies [52].

Many limitations are the same as those of approach 5 (IAS) and include the lack of some data sources available on QHINs (eg, payers and many small EHRs are not participating), performance issues when trying to query large geographic areas, and imperfect record matching resulting in missed data.

However, the major limitation is that, unlike the queries in approach 5, support for research queries is not currently a requirement for TEFCA participants, and the effort required to make research a purpose of use that is answered reliably by sites is substantial. SMEs described how participating providers and EHR vendors would require processes to be in place to ensure that a query is legitimate (eg, participants are consented, the study is IRB approved), and an enforcement mechanism to ensure that the data are used only for the designated purpose and in compliance with HIPAA regulations. This will be challenging because rules governing consent for such queries vary across states. Some have proposed that QHINs offer digital identity services to patients and allow them to control and view access to their data—by putting the patient in charge, this would obviate trust issues among network participants [53]. Yet, one SME suggested that research queries “may never happen at scale.”

SMEs described how national queries were being used for research today. Some existing companies query these networks for treatment purposes and then use the data for research as a secondary use. For example, one company justified its use of the treatment purpose of use by establishing itself as a telemedicine provider that helps patients navigate the health system, and then it uses those data for research with participant consent. In other cases, some forms of clinical trials might be considered both treatment and research. SMEs hoped that a future legal pathway for research queries would clarify this situation.

### Aggregate Data Source

#### Description

In this approach, the participant consents to allowing the research team to collect data from a company that has aggregated datasets from multiple providers (directly or through EHR vendors or national laboratories) and/or payers (directly or through claims clearinghouses or pharmacy aggregators). The company serves as a marketplace connecting research teams with the data sources. The research team works with the organization to specify the data they need and pays a fee. The organization handles all the governance, business, and operational issues, including securing approval from the data sources, identifying the records associated with the consented patients, and preparing analytic datasets.

#### Assessment

This approach is available today, widely accessible to all participants regardless of their ability to use technology, involves minimal burden on the participant, and allows research teams to control the timing of the queries to reduce variability in retrieved data. Limitations include cost to the research team, limits in the extent of available data sources, and lack of patient control over or awareness of the data available. Many data sources (eg, providers and payers) will only agree to release data anonymously. Research teams conducting studies with consented patients could meet this requirement by bifurcating their team into those with access to limited identifiable data (eg, for recruiting) and those with access to deidentified data for analyses. This may add burden to the study team and may not be feasible for some research teams.

## Discussion

### Principal Findings

We identified 8 approaches that would facilitate research team access to data from multiple providers and payers for participants in US studies, and 12 evaluation criteria. There were clear benefits and limitations for each approach. Participant-mediated approaches have the major advantage of bypassing the need for complex governance and privacy management processes, but they burden the participant, require effort or costs for the study team, and have incomplete connectivity to data sources, especially payers. Approaches involving queries of data exchange infrastructure are robust in some regions but vary substantially in terms of the data available and their ability to support research, and they may be challenging to implement at a national scale. Aggregate data sources allow for queries of national-level data in a way that addresses privacy management and governance issues but have limits in the scope of data sources. The benefits and limitations of each approach will likely change as the landscape evolves, driven largely by public policy.

In addition to the three steps of our focus (identifying the location of the data, retrieving the data, and sharing the data with research studies), SMEs emphasized the value of a fourth step: preparing the data for research. Many SMEs emphasized the value of these services, including normalization, conformance testing, and quality checks. As FHIR is a relatively new format for health data, most researchers are not accustomed to analyzing it. Many researchers may benefit from working with organizations familiar with the data’s idiosyncrasies, and that can produce an analytic dataset tailored to the needs of the research project. There are active efforts to make FHIR data easier to validate [54] and analyze [55], which may mitigate this concern.

The value of synergies between payer and provider data (eg, leveraging payer data to identify and retrieve data from providers that a participant has visited previously) is only beginning to be explored and has the potential to provide a feedback loop in this process. For example, payer data can identify gaps in provider data and locations to target for data collection.

This complex landscape is the result of the fragmented structure of the US health care system, which produces the data used in research, wherein patients receive care from many different providers who use different EHR systems and are covered by different payers. Within this landscape, each research study will need to consider the best option to address its data needs within study budgets. Studies may consider multiple approaches to obtaining as complete a dataset as possible. For example, a study might use a participant-mediated approach for Medicare claims data in combination with working with a local HIE or aggregate data source for EHR data.

### Contribution

To our knowledge, this is the first study attempting to describe the US landscape of approaches to collect data from multiple providers and payers for research. Our results provide a comprehensive comparison of the benefits and limitations of each approach, allowing research teams to understand existing and anticipated future options and policymakers to target areas for improvement.

We found only 1 publication that discusses how a participant-mediated approach was used to collect data for a study [41]. Other studies may have used this approach but have not described or assessed it. Sayeed et al [37] assessed the potential for using FHIR standards for multiple research tasks, including collecting EHR data, as part of the People Heart Study. We did not find any assessments of the use of TEFCA IAS. HIEs and aggregated data sources have been used for studies involving consented patients, but we did not find a systematic examination of these approaches.

### Limitations

This study was limited to a US focus; the landscape in other countries regarding the collection of comprehensive data for research will likely differ and would be worth investigating in future work. Our data relied on SMEs and may not have included all relevant perspectives. The landscape of approaches is rapidly evolving, and some of our findings may become outdated, especially as new policies are implemented. Additionally, we did not conduct a systematic literature review, as our primary objective was to gather expert views to inform our analysis. However, we did review relevant literature identified by the research team and SMEs, who likely were aware of influential publications. Our scope was limited to one use case: the collection of provider and payer data for consented participants. We excluded other study workflows (eg, recruitment) and other types of studies (eg, population-level analyses of deidentified data).

### Conclusions

Complex and evolving trade-offs exist among the approaches we identified for collecting comprehensive electronic health data for research in the United States. We did not find a clear “winner”—the optimal approaches will likely vary considerably based on factors specific to a study. Continued development, exploration, and evaluation of all approaches are warranted.

Policies supporting participant-mediated exchange could greatly improve those approaches by making FHIR endpoints more findable (eg, through a national public directory), making it easier for researchers to broadly register an app (eg, requiring a central location for all of an EHR vendor’s customers), and ensuring that FHIR APIs are conformant with standards (eg, automated testing). Conformance requirements for FHIR APIs and data standards are particularly needed for private payer endpoints and are more efficient than expecting each research team to deal with the data issues individually. Advances in digital identity and the use of SSO across providers and patients may facilitate portal signup and the ability for study participants to sign in and pull records. Ongoing conformance evaluation of FHIR endpoints and assessments of participant ability to find and share data from prior sites of care would help inform improvement efforts.

Policies may also support queries of the data exchange infrastructure. Some regional HIEs have demonstrated the feasibility and value of using their existing infrastructure to pull data for research [50,56-58]. These HIEs have distinct advantages, such as coverage of most relevant local data sources, familiarity with state and local privacy laws, established governance processes, and knowledge of their members’ data quality issues. Some are more advanced in supporting research than others, and there is room for additional innovation. Policies could support establishing HIEs in regions that lack them, developing more capabilities to support research, and facilitating the sharing of best practices and evaluation. National efforts to make data available for research are still in their infancy, and it is not clear if or how the trust issues among data holders can be resolved, especially considering recent litigation [59,60]. The IAS may be the most promising approach that uses the national networks, but it will require clarifying the liability rules for data holders when they respond to requests. Evaluations should assess retrieved data in comparison with data obtained using a treatment purpose of use.

As these approaches evolve, ongoing rigorous evaluation and benchmarking of the benefits and limitations of each approach would empower research teams to make better decisions for study data collection.

## Supplementary material

10.2196/86330 Multimedia Appendix 1 Evaluation of approaches to collecting comprehensive provider and payer data for research.

10.2196/86330 Checklist 1 SRQR checklist.

## Abbreviations

API: application programming interface
CMS: Centers for Medicare & Medicaid Services
EHI: electronic health information
EHR: electronic health record
FHIR: Fast Health Interoperability Resources
HIE: health information exchange
HIPAA: Health Insurance Portability and Accountability Act
IAS: Individual Access Service
IRB: Institutional review board
QHIN: qualified health information network
SMART: Substitutable Medical Applications and Reusable Technologies
SME: subject matter expert
SSO: single sign-on
TEFCA: Trust Exchange Framework and Common Agreement

## References

[1] (2022). Adoption of electronic health records by hospital service type 2019-2021. Office of the National Coordinator for Health Information Technology.

[2] Riva A, Mandl KD, Oh DH (2001). The personal internetworked notary and guardian. Int J Med Inform.

[3] Al-Sahab B, Leviton A, Loddenkemper T, Paneth N, Zhang B (2024). Biases in electronic health records data for generating real-world evidence: an overview. J Healthc Inform Res.

[4] Gurupur V, Hooshmand S, Prabhu DF, Trader E, Salvi S (2025). Incompleteness of electronic health records: an impending process problem within healthcare. Healthcare (Basel).

[5] Kar S, Bessette LG, Wyss R, Kesselheim AS, Lin KJ (2023). The impact of electronic health record discontinuity on prediction modeling. PLoS One.

[6] Martin S, Wagner J, Lupulescu-Mann N (2017). Comparison of EHR-based diagnosis documentation locations to a gold standard for risk stratification in patients with multiple chronic conditions. Appl Clin Inform.

[7] Advancing nationwide interoperability with TEFCA®. Office of the National Coordinator for Health Information Technology.

[8] Black JR, Hulkower RL, Ramanathan T (2018). Health information blocking: responses under the 21st Century Cures Act. Public Health Rep.

[9] Lye CT, Forman HP, Daniel JG, Krumholz HM (2018). The 21st Century Cures Act and electronic health records one year later: will patients see the benefits?. J Am Med Inform Assoc.

[10] Majumder MA, Guerrini CJ, Bollinger JM, Cook-Deegan R, McGuire AL (2017). Sharing data under the 21st Century Cures Act. Genet Med.

[11] Gordon WJ, Rudin RS (2022). Why APIs? Anticipated value, barriers, and opportunities for standards-based application programming interfaces in healthcare: perspectives of US thought leaders. JAMIA Open.

[12] Kahn MG, Raebel MA, Glanz JM, Riedlinger K, Steiner JF (2012). A pragmatic framework for single-site and multisite data quality assessment in electronic health record-based clinical research. Med Care.

[13] Pham HH, Schrag D, O’Malley AS, Wu B, Bach PB (2007). Care patterns in Medicare and their implications for pay for performance. N Engl J Med.

[14] Pham HH, O’Malley AS, Bach PB, Saiontz-Martinez C, Schrag D (2009). Primary care physicians’ links to other physicians through Medicare patients: the scope of care coordination. Ann Intern Med.

[15] Fang H, Frean M, Sylwestrzak G, Ukert B (2022). Trends in disenrollment and reenrollment within US commercial health insurance plans, 2006-2018. JAMA Netw Open.

[16] Szolovits P, Long WJ, Kohane I, Pauker SG (1994). Guardian angel: patient-centered health information systems. https://dspace.mit.edu/entities/publication/f1e3abf1-73d7-4c08-8851-b23bf5e0bcba.

[17] Yasnoff WA, Shortliffe EH (2014). Lessons learned from a health record bank start-up. Methods Inf Med.

[18] Everson J, Chang W, Patel V, Adler-Milstein J (2024). The state of health information organizations and plans to participate in the federal exchange framework. Health Aff Sch.

[19] Chimanuka Murhima’alika C, Mary M, Chiribagula Zalinga C (2025). Exploring opportunities to strengthen maternal and perinatal death surveillance and response: a landscape analysis of surveillance and health information systems in the Eastern Democratic Republic of Congo. Confl Health.

[20] Meek JY, Nelson JM, Hanley LE, Onyema-Melton N, Wood JK (2020). Landscape analysis of breastfeeding-related physician education in the United States. Breastfeed Med.

[21] Shalash A, Nemer M, Abu-Rmeileh N, Kittaneh M, Kelly D, Elmusharaf K (2025). Mapping stakeholders, services, data, and the information system for adolescent health in the West Bank. Reprod Health.

[22] Hsieh HF, Shannon SE (2005). Three approaches to qualitative content analysis. Qual Health Res.

[23] Berg BL (2007). Qualitative Research Methods for the Social Sciences.

[24] Saunders B, Kitzinger J, Kitzinger C (2015). Anonymising interview data: challenges and compromise in practice. Qual Res.

[25] (2015). Medicare and Medicaid programs; Electronic Health Record Incentive Program-Stage 3 and modifications to meaningful use in 2015 through 2017. Federal Register.

[26] (2025). CMS seeks public input on improving technology to empower medicare beneficiaries. CMS.

[27] (2025). Health tech ecosystem categories. CMS.

[28] Rudin RS, Hillestad R, Ridgely MS, Qureshi NS, Davis JS, Fischer SH (2019). Defining and evaluating patient-empowered approaches to improving record matching. Rand Health Q.

[29] (2025). Tiered OAuth for user authentication. HL7.

[30] (2023). CARIN Alliance and HHS release digital identity federation report about their test proof of concept. Leavitt Partners.

[31] Interoperability framework. CMS.

[32] (2024). User-access brands and endpoints. HL7.

[33] (2024). Health data, technology, and interoperability: certification program updates, algorithm transparency, and information sharing. Federal Register.

[34] (2025). CMS building foundational infrastructure for digital healthcare ecosystem. Centers for Medicare & Medicaid Services.

[35] (2026). HealthKit. Apple Developer.

[36] (2026). Medical records. Android Developers.

[37] Sayeed R, Kreda D, Mandel JC (2025). A standards-based approach to digital health research: implementing the people heart study. J Am Med Inform Assoc.

[38] Truslow J, Spillane A, Lin H (2024). Understanding activity and physiology at scale: the Apple Heart & Movement Study. NPJ Digit Med.

[39] SHL protocol specification. SMART Health Links.

[40] Keeler B (2021). Indiana Jones and the personal health record. Health API Guy.

[41] Krumholz HM, Sawano M, Bhattacharjee B (2025). The PAX LC trial: A decentralized, phase 2, randomized, double-blind study of nirmatrelvir/ritonavir compared with placebo/ritonavir for long COVID. Am J Med.

[42] Standardized data: the OMOP common data model. Observational Health Data Sciences and Informatics.

[43] Procure-wip. GitHub.

[44] (2025). Homepage. RTI Wellmine.

[45] (2020). 21st Century Cures Act: interoperability, information blocking, and the ONC Health IT Certification Program. Federal Register.

[46] Code of conduct. CARIN Alliance.

[47] Kulatunga J (2025). TEFCA IAS - deep dive into patient matching & RLS responses. Fasten Health Blog.

[48] Health data utilities. CIVITAS: Networks for Health.

[49] Gryczynski J, Nordeck CD, Welsh C, Mitchell SG, O’Grady KE, Schwartz RP (2021). Preventing hospital readmission for patients with comorbid substance use disorder: a randomized trial. Ann Intern Med.

[50] Clinical data drives insight and innovation to transform our healthcare system. CRISP Health.

[51] EHR Good Neighbor. Scaling research data access statewide with bulk FHIR.

[52] Resource subscription. HL7.

[53] Mandel JC, Pollak JP, Mandl KD (2022). The patient role in a federal national-scale health information exchange. J Med Internet Res.

[54] Qualifier. GitHub.

[55] SQL on FHIR. HL7 FHIR Implementation Guides.

[56] Homepage. HealthShare Exchange (HSX).

[57] How we bring the data to you. Regenstrief Institute.

[58] Homepage. MyHealth Access Network.

[59] Adler S (2026). Epic sues health information exchange network alleging improper record access. The HIPAA Journal.

[60] Keeler B (2024). Epic v. particle 2: the problem of secondary use. Health API Guy.

