# Supplementary material for: Approaches to Collect Comprehensive Electronic Patient Data Across Multiple Providers and Payers for Research: Landscape Analysis
Source: J Med Internet Res. 2026 Jun 15;28:e86330. doi: 10.2196/86330 (PMC13268630; doi:10.2196/86330)
Supplement: Checklist 1 [file jmir-v28-e86330-s002.pdf]

**Standards for Reporting Qualitative Research (SRQR) Checklist**

http://www.equator-network.org/reporting-guidelines/srqr/

| | Item Description | Location (or reason for not reporting) |
|---|---|---|
| **Title & Abstract** | | |
| Title | Describe the nature and topic of the study. Identify the study as qualitative or indicate the approach or data collection methods. | 1 |
| Abstract | Summarise the key elements of the study using the abstract format of the intended publication. | 1 |
| **Introduction** | | |
| Problem Formulation | Describe the problem/phenomenon studied, its significance, relevant theory and empirical work, and gaps in current knowledge. | 3-4 |
| Purpose or research question | Describe the purpose of the study and specific objectives or questions. | 4 |
| **Methods** | | |
| Qualitative approach and research paradigm | Describe your qualitative approach, your guiding theory (if appropriate), and research paradigm, and reasons for your choices. | 5-6 |
| Researcher characteristics and reflexivity | Describe how researchers' characteristics may influence the research, including personal attributes, qualifications/experience, relationship with participants, assumptions, and/or presuppositions; potential or actual interaction between researchers' characteristics and the research questions, approach, methods, results and/or transferability. | 5-6 |
| Context | Describe the setting/site(s) in which the study was conducted, why it was selected, and any other salient contextual factors that may influence the study. | 5 |

| | | |
|---|---|---|
| Sampling strategy | Describe how and why research participants, documents, or events were selected; criteria for deciding when no further sampling was necessary, and the rationale for those criteria. | 5 |
| Ethical issues pertaining to human subjects | Describe any approval by an appropriate ethics review board and participant consent, or explain any lack thereof. Describe any other confidentiality and data security issues. | 6 |
| Data collection methods | Describe the types of data collected; details of data collection procedures including (as appropriate) start and stop dates of data collection and analysis, iterative process, triangulation of sources/methods, and modification of procedures in response to evolving study findings. Describe your rationale for these choices. | 5-6 |
| Data collection instruments and technologies | Describe any instruments (e.g., interview guides, questionnaires) and devices (e.g., audio recorders) used for data collection; describe if/how the instrument(s) changed over the course of the study. | 5 |
| Units of study | Describe the number and relevant characteristics of participants, documents, or events included in the study. Describe the level of participation. | 6-7 |
| Data processing | Describe the methods for processing data prior to and during analysis, including transcription, data entry, data management and security, verification of data integrity, data coding, and anonymisation / deidentification of excerpts. | 6 |
| Data analysis | Describe the process by which inferences, themes, etc. were identified and developed, including the researchers involved in data analysis; usually references a specific paradigm or approach. Describe why you chose this process. | 6 |
| Techniques to enhance trustworthiness | Describe any techniques to enhance trustworthiness and credibility of data analysis,(e.g., member checking, | 6 |

| | | |
|---|---|---|
| | triangulation, audit trail). Describe why you chose these techniques. | |
| **Results** | | |
| Synthesis and interpretation | Describe the main findings (e.g., interpretations, inferences, and themes); might include development of a theory or model, or integration with prior research or theory. | 6-12 |
| Links to empirical data | Provide evidence (e.g., quotes, field notes, text excerpts, photographs) to substantiate analytic findings. | 12-19 |
| **Discussion** | | |
| Integration with prior work, implications, transferability, and contribution(s) to the field | Summarize the main findings, explain how findings and conclusions connect to, support, elaborate on, or challenge conclusions of earlier scholarship; discuss the scope of application/generalizability; identify unique contribution(s) to scholarship in a discipline or field. | 19-20 |
| Limitations | Discuss the trustworthiness and limitations of findings | 21 |
| **Other** | | |
| Conflicts of interest | Describe any potential sources of influence or perceived influence on study conduct and conclusions. Describe how these were managed. | 22 |
| Funding | Describe sources of funding and other support. Describe the role of funders in data collection, interpretation, and reporting. | 22 |
